# Be optimistic or be cautious? Affective forecasting bias in allocation decisions and its effect

**DOI:** 10.3389/fpsyg.2022.1026557

**Published:** 2022-12-13

**Authors:** Lei Liu, Wujun Sun, Ping Fang, Yuan Jiang, Li Tian

**Affiliations:** ^1^School of Labor and Human Resources, Renmin University of China, Beijing, China; ^2^Faculty of Education, Henan Normal University, Xinxiang, China; ^3^College of Psychology, Capital Normal University, Beijing, China; ^4^College of Psychology, Beijing Sport University, Beijing, China

**Keywords:** affective forecasting, affective forecasting bias, allocation decisions, ultimatum game, generosity, well-being

## Abstract

**Introduction:**

People’s forecasts of their future emotions play an essential role in their behavior and experience of well-being. However, their emotional reactions may fall short of what they expect, which has implications for subsequent decision making. The current paper investigated the accuracy of affective forecasting about resource allocations and how this (in)accuracy predicts future allocation decisions.

**Methods:**

Two experimental studies were conducted. Study 1 (*N* = 84) examined the extent to which people can accurately predict how allocation decisions will feel using an ultimatum game on the part of the allocator. Study 2 tested whether the affective forecasting bias affects future allocation decisions, with 192 participants playing a two-round ultimatum game on the part of allocators.

**Results:**

Study 1 found an affective forecasting bias, and people anticipated more powerful emotional reactions to both positive and negative allocation events than they actually experienced when the events occurred. Study 2 found that increased affective forecasting bias resulted in less generous decisions in positive event conditions and more generous decisions in negative event conditions.

**Discussion:**

These results extend previous findings concerning affective forecasting bias and the feelings-as-information model in resource allocation interactions and show that the difference between anticipated and experienced emotion is also informative in allocation decisions. The results suggest that being more cautious when forecasting positive outcomes and more optimistic when forecasting negative outcomes can be beneficial to one’s well-being.

## Introduction

Decision making is a vital part of life. People are faced with a variety of choices every day, whether it is a daily chore like ordering a meal or a major issue like choosing whom to marry. We often need to choose from many options, and different decisions often mean different directions in life and development, thus making good decisions is key to our future well-being (Lerner et al., 2015). Existing research suggests that people often make the most favorable decisions based on their anticipated emotions about different outcomes (Gilbert et al., 1998; Wilson and Gilbert, 2003, 2005). The quality of decisions rests heavily on the accuracy of people’s forecasting of future emotions (Greene et al., 2016; Lench et al., 2019). Unfortunately, a large body of research has indicated that anticipated emotions and actually experienced emotions are often inconsistent, and this bias (inaccuracy) can also have important implications for subsequent decisions, resulting in complex human decision-making behaviors (Hoerger et al., 2016; Levine et al., 2018). While research has discussed the impact of affective forecasting and the effect the anticipated emotions it generates on decision making, the prevalence and role of affective forecasting bias in social decision making, especially allocation decision making, has not received sufficient attention and needs to be further explored.

In the following sections, we reviewed the affective forecasting bias research and used the feelings-as-information model to explore the effect of affective forecasting bias on allocation decisions. Study 1 tested whether affective prediction bias is present in resource allocation, and study 2 examined whether and when affective forecasting bias influences subsequent allocation decisions. This study makes contributions to the existent literature in three folds. First, it provides experimental evidence for the prevalence and role of affective forecasting bias in allocation decision making and enriches academic knowledge and understanding of this concept; Second, it provides a new emotional perspective—affective forecasting bias—in understanding and studying individuals’ resource allocation decisions; Third, it extends previous findings concerning the feelings-as-information model to resource allocation. Finally, the limitations of this study and directions for future research are discussed.

### Affective forecasting bias and decision making

People always forecast what their future emotional responses will be to both positive and negative events when making a decision (Wilson and Gilbert, 2003, 2013; Buechel et al., 2014). That is, people are involved in affective forecasting (Wilson and Gilbert, 2003). The outcome of the forecasting is anticipated emotion, which is the ‘cognitive’ and future-oriented emotion experienced when anticipating a future outcome (Baumgartner et al., 2008).

In the past decades, a substantial body of research has addressed the question of how well people predict their affective reactions to future events. Research has shown that people often correctly predict the valence of their emotional responses and the specific emotions they will experience, but they are not very good at correctly predicting their initial intensity of the responses and the duration of emotion (for reviews, see Wilson and Gilbert, 2003). People anticipate feeling worse after negative events and better after positive events than they actually experience when the events occur (e.g., Wilson et al., 2000; Gilbert et al., 2004). This tendency to overestimate the intensity of future emotion has been termed impact bias (Wilson and Gilbert, 2003; Dillard et al., 2021). For instance, Buehler and Mcfarland (2001) have confirmed this basic forecasting bias in five experimental studies in which participants overestimated the intensity of their affective reactions across a wide variety of life events (e.g., trips and vacations, major purchases, family visits, academic failures, dental appointments). Other studies have also shown that when people predicted they would fail to receive tenure (Gilbert et al., 1998), break up (Eastwick et al., 2008), participate in a talent show (Feys and Anseel, 2015), win or lose games (Lau et al., 2016), engage in passive activities (Schiffer and Roberts, 2018), be exposed to opposing views (Dorison et al., 2019), or participate in policymaking (Cohen-Blankshtain and Sulitzeanu-Kenan, 2021), they tended to expect stronger affective reactions than they actually experienced. To explore this phenomenon further, scholars have operationally defined affective forecasting bias as the difference between anticipated and experienced affect (Patrick et al., 2007; Lau et al., 2016).

Research has shown that affective forecasting bias plays a complicated role in decision-making (Levine et al., 2018). Bias in affective forecasting may prove costly and lead to inappropriate decisions. For instance, Wilson and Gilbert (2005) found that couples decided to break up due to the false prediction that staying in the relationship would bring greater emotional harm. People were also less likely to ask for genetic test results if they expected to be distressed by them (Ferrer et al., 2015). In another study, women who expected to experience greater stress were more likely to refuse medications after they received advice to take them to reduce their high risk of breast cancer (Hoerger et al., 2016). Rehabilitation experts overestimated the intensity and duration of a physical injury, a bias that could result in unfair court judgments granting more compensation than necessary (Greene et al., 2016). However, affective forecasting bias may also serve a functional purpose. Affective forecasters may strategically overestimate the hedonic impact of events to motivate themselves to produce the events that they forecast (Morewedge and Buechel, 2013).

### Affective forecasting bias and allocation decisions

In everyday life, people are often involved in the allocation of limited resources (e.g., money or time) between themselves and others, which is referred to as allocative decision making (Rilling and Sanfey, 2011; van der Schalk et al., 2015). As an essential type of social decision-making, allocative decisions involve psychological conflicts between self-interest and the interests of others and may reflect either prosocial or selfish preferences (van Dijk and De Dreu, 2021). Decisions about resource allocation are important, for example, when deciding how to allocate salary raises from a fixed pool or health care resources among patients with different needs (Rilling and Sanfey, 2011; Argyris et al., 2022). Furthermore, these allocation decisions are essential for individual social interaction, interpersonal relationships, and well-being, and therefore have an important place in decision-making (Rilling and Sanfey, 2011). In this study, we extend affective forecasting research by examining the degree of forecasting accuracy of individuals’ future emotional responses to allocation decisions.

Research has shown that observers’ anticipated emotions influence their resource allocations and that people may tend to make allocation decisions based on affective forecasting (van der Schalk et al., 2015). When making decisions, they often imagine how they might feel about the consequences of the allocation and use their anticipated emotions to guide their choices (Nelissen et al., 2011; van der Schalk et al., 2012, 2015). Bargaining offers in ultimatum game experiments are guided by the emotions that proposers anticipate when considering their offers, and findings suggest that both anticipated fear and anticipated guilt can increase proposers’ ultimatum offers (Nelissen et al., 2011). Van der Schalk et al. (2012) have found that when participants expected to be proud of their fair allocation, they bid more to anonymous others, and when they expected to regret their fair allocation, they bid less to anonymous others. There is an implied assumption in these studies that people are accurate in their anticipated emotions, but this has not been found to be the case (Wilson and Gilbert, 2003; Patrick et al., 2007; Lau et al., 2016). Affective forecasting bias may also be prevalent and an important influencing factor on the decision-making process in allocation decisions. Previous studies have not included affective forecasting bias as a key research variable to be examined.

In summary, affective forecasting plays an important role in decision making, as does affective forecasting bias. The affective forecasting bias can result in individuals making inappropriate decisions, but it may also confer some benefit. The effect of affective forecasting bias on decision-making may vary in different contexts. As an important type of social decision-making, allocative decisions are associated with prosocial or selfish preferences and important for individual social interaction, interpersonal relationships, and well-being. Unfortunately, whether affective forecasting bias leads individuals to be more or less generous when allocating limited resources has received little research attention. As a result, in this paper, we extended affective forecasting research by examining the degree of forecasting accuracy of individuals’ future emotional responses to allocation decisions and its effect.

The ultimatum game has been used in most social interaction studies (Güth and Kocher, 2014; Tisserand et al., 2015). The game is developed by Güth et al. (1982) and has two players anonymously dividing a certain amount of money. The allocator first offers a proportion of the money to the recipient, and the recipient chooses to accept or refuse the offer. If the recipient accepts the offer, the money is distributed according to the allocator’s offer. On the other hand, if the responder refuses the offer, neither receives any money. The ultimatum game, therefore, models decisions about resource allocation on the part of the allocator (Rilling and Sanfey, 2011; van der Schalk et al., 2015). Thus, the ultimatum game on the part of the proposer is very suitable for studying resource allocation decisions.

### The current studies

In this paper, we present two experimental studies that empirically examined whether there is an affective forecasting bias in resource allocation, and whether affective forecasting bias have an influence on subsequent allocation decision-making by using the ultimatum game paradigm.

## Study 1

The purpose of study 1 was to assess the degree of accuracy or bias in individuals’ affective forecasts for allocation decisions using an ultimatum game on the part of the allocator. We examined both positive and negative events and outcomes in the same context. In line with previous forecasting bias research, we expected that people’s predictions of emotional outcomes for resource allocation would be inaccurate: people would overestimate how good they would feel about a positive event and how bad they would feel about a negative event.

### Materials and methods

#### Participants

The participants were 84 undergraduate student volunteers (44 male and 40 female) enrolled in introductory psychology classes at a university in central China who received course credit for participating. Their mean age was 19.14 years (*SD* = 1.18), and none had participated in the affective forecasting experiment or the ultimatum game before.

#### Materials

##### Adapted ultimatum game

In our adapted ultimatum game, two players have to distribute 100 RMB (about 15.40 US dollar) between them, having nine options (¥10–¥90, ¥20–¥80, ¥30–¥70, ¥40–¥60, ¥50–¥50... ¥80–¥20, ¥90–¥10). The allocator decides how to distribute the money, and the recipient can accept or reject this distribution. If the recipient accepts the allocation, then they will get the money according to this distribution, and if the recipient rejects it, neither of them gets any money. The ultimatum game, therefore, models decisions about resource allocation on the part of the allocator. Thus, in this study, all participants were allocators. In addition, according to the classical experimental paradigm of affective forecasting bias, generally, researchers consider both positive and negative outcomes (Buehler and 2001; Pauketat et al., 2016). The recipients were not arranged in order to manipulate the results of the recipients, i.e., half of the participants accepted the allocator’s offer, and half of the participants rejected the allocator’s offer, thus generating both positive and negative event conditions.

##### Emotional intensity scale

The emotional intensity scale referred to previous research on affective forecasting bias and was adapted according to the experimental context of this study (Pauketat et al., 2016). The Positive Emotional Intensity Scale includes three positive emotional adjective words (happy, cheerful, and glad), ranging from 1 (not at all) to 9 (very much), and asks the participants to report the intensity of the three emotions, which are then averaged to produce a single indicator of positive emotional intensity (Cronbach’s a = 0.86). The Negative Emotional Intensity Scale includes three negative emotional adjective words (sad, depressed, and down), ranging from 1 (not at all) to 9 (very much), and asks the participants to report the intensity of the three emotions, which are then averaged to produce a single indicator of negative emotional intensity (Cronbach’ s a = 0.84).

#### Procedure

The participants were invited to the laboratory, with computers presenting the E-prime 2.0 version of the ultimatum game. The participants first filled in the basic information, including their gender, age, and initial emotion. Participants were then informed that they would be paired with one of the other participants who were at the same time in another room of the laboratory and play an allocation game on the computer. They were told they were assigned as an allocator or recipient based on a chance procedure. After providing consent, the experimenter explained to the participants in detail how to play the ultimatum game, with the following specific instructions: “You will complete this experiment with a student from another room. In this experiment, you will jointly allocate $100. You, as the allocator, will choose one of nine allocation options (allocate ¥10, ¥20, ¥30, ¥40, ¥50, ¥60, ¥70, ¥80, ¥90) to the other student. The other student, as the recipient, will decide whether to accept or reject the allocation option. For example, if you allocate ¥10 to the other student, the other student will receive ¥10, and you will receive ¥90. And so on. If the recipient accepts the option, the two of you will distribute the money according to this option. If the recipient rejects the option, neither of you will receive the money. At the end of the experiment, you will be paid the appropriate percentage based on your distribution in the experiment.” In this experiment, all participants were allocators; That is, there was no another recipient in the other room.

Next, participants played the ultimatum game. Whatever the allocator offers, we hypothesize that if his offer is rejected, he does not get a penny, which is a negative event for him. If the other student accepts his offer, he gets the corresponding amount of money, which is a positive event for him. We test this hypothesis in a pilot study. The results indicated that offer acceptance could induce positive emotions and rejection could induce negative emotions (see Table 1). We could thus safely consider the rejection of their offer to be a negative event and the acceptance of their offer to be a positive event. Forty-two participants were randomly assigned to a positive event condition (recipients accepted the allocation) and 44 participants were randomly assigned to a negative event condition (recipients rejected the allocation).

**Table 1:** Descriptive statistics and *t*-test of emotion valence for offer acceptance and rejection.

	The offers were accepted	The offers were rejected			
	(*M* ± *SD*)	(*M* ± *SD*)	*t*	*p*	Cohen’s *d*
Predicted positive emotion	7.04 ± (0.96)	1.91 ± (0.98)	15.11	0.000	0.94
Predicted negative emotion	1.91 ± (0.98)	6.77 ± (1.29)	−16.4	0.000	0.90
Experienced positive emotion	6.86 ± (1.28)	2.01 ± (1.14)	15.49	0.000	0.89
Experienced negative emotion	1.66 ± (0.64)	6.36 ± (1.48)	−15.99	0.000	0.90

*N* = 60.

##### Affective forecast

All participants predicted their emotional reactions to both outcomes before playing the game. The instructions are, “Please imagine that you and the student in another room are working together on this experiment. You, as the allocator, have chosen one of the nine allocation options (allocate ¥10, ¥20, ¥30, ¥40, ¥50, ¥60, ¥70, ¥80, ¥90) to the other student.” In a balance manner, half of the participants were first asked to imagine that the offer was accepted, that they got the money accordingly, and to rate their anticipated emotion on the positive emotional intensity scale. Then they were asked to imagine that the offer was rejected, they got nothing, and to rate their anticipated emotion on the negative emotional intensity scale. The other half of the participants made their forecasting in the opposite order.

After a 30-min break, participants were asked to propose the exact offers as their imagined offers to other students. The offers were rated on a 9-point scale, with 1 representing 10 RMB, 2 representing 20 RMB, 3 representing 30 RMB, and 9 representing 90 RMB.

##### Affective experience

In the positive event condition, participants received feedback that their offers were accepted, and they got the money accordingly. They then rated their experienced emotion on the positive emotional intensity scale. In the negative event condition, on the other hand, participants received feedback that their offers were rejected, and they got nothing. Then they rated their experienced emotion on the negative emotional intensity scale.

At the end of the session, all participants were debriefed about the study’s objectives. No one had doubts about the authenticity of the interaction. It was explained that they all had played the role of the allocator. Subsequently, the participants were paid according to the results of their distribution. All materials and procedures were approved by the Institutional Review Board (IRB) of the authors’ institution.

### Results

To make within-subject comparisons of anticipated and experienced emotion, we first determined whether each participant’s offer was accepted (positive outcome) or rejected (negative outcome). We then selected the affective forecasting that corresponded to each outcome. Following the method employed by Sevdalis and Harvey (2007), affective forecasting bias of individual participants was calculated by subtracting their ratings of experienced emotions from those of anticipated emotions.

#### Accuracy of positive event forecasting

Forecasters accurately believed that their offers being accepted would make them happy, but they were wrong about how happy they would be. Overall, as shown in Table 2, participants anticipated experiencing high levels of positive affect about a positive outcome (*M* = 7.05, *SD* = 0.92). However, their experiences of the positive outcome proved to be substantially less positive than they anticipated (*M* = 6.35, *SD* = 1.04). A paired samples *t*-test showed that this difference was statistically significant, *t* (41) = 4.17, *p* = 0.000, mean difference = 0.70, Cohen’s *d* = 0.64. Thus, participants clearly over-estimated their positive reactions to the positive outcome. In short, forecasters’ estimates of their affective reactions to a positive allocation decision showed evidence of affective forecasting bias.

**Table 2:** Descriptive statistics and *t*-test for anticipated and experienced emotions.

		Anticipated emotion	Experienced emotion	Affective forecasting bias			
	*n*	(*M* ± *SD*)	(*M* ± *SD*)		*t*	*p*	Cohen’s *d*
*Study 1*							
Positive event	42	7.05 ± (0.92)	6.35 ± (1.04)	0.70	4.17	0.000	0.64
Negative event	42	6.29 ± (1.25)	5.78 ± (1.46)	0.51	2.19	0.034	0.34
*Study 2*							
Positive event	96	6.94 ± (1.09)	6.34 ± (1.63)	0.60	4.64	0.000	0.47
Negative event	96	6.01 ± (1.80)	5.52 ± (1.86)	0.49	4.01	0.000	0.41

*d* = 0.2 (small effect); *d* = 0.5 (medium effect): *d* = 0.8 (large effect).

#### Accuracy of negative event forecasting

Forecasters also accurately believed that their offers being rejected would make them unhappy, but they were wrong about how unhappy they would be. Overall, as shown in Table 2, participants anticipated experiencing high levels of negative affect about a negative outcome (*M* = 6.29, *SD* = 1.25). However, their experiences of the negative outcome proved to be substantially less negative than they anticipated (*M* = 5.78, *SD* = 1.46). A paired samples *t*-test showed that this difference was statistically significant, *t* (41) = 2.19, *p* = 0.034, mean difference = 0.51, Cohen’s *d* = 0.34. Thus, participants clearly over-estimated their negative reactions to the negative outcome. In short, individuals’ forecasts of their emotional reactions to a negative allocation decision showed evidence of affective forecasting bias.

### Discussion

The first study examined affective forecasting bias in resource allocation, and is the first study to examine the bias as it relates to the decision making of resource allocation. In line with previous research, people anticipated more powerful reactions to both positive and negative allocation events than they ended up feeling. This study supports our hypotheses concerning “affective forecasting bias” in people’s forecasting of their affective reactions to resource allocation events, and provides a good basis for continuing to explore its impact on subsequent allocation decisions. In study 2, we examined the relationship between this biased affective forecasting and subsequent resource allocation decisions in the same ultimatum game.

## Study 2

The purpose of study 2 was to examine the effect of the affective forecasting bias on subsequent allocation decision making. As study 1 showed, in the positive event condition, the affective forecasting bias showed that the intensity of anticipated positive emotions was higher than the actually experienced one, resulting in feeling worse than forecasted. In the negative event condition, the affective forecasting bias also showed that the intensity of anticipated negative emotions was higher than the experienced one, resulting in feeling better than forecasted.

The feelings-as-information model proposes that in order to make decisions, people ask themselves “how do I feel about it?.” This model asserts that positive feelings inform a safe and benign environment that does not require careful detailed processing, and that individuals rely on heuristic, top-down process. In contrast, negative feelings suggest the presence of a problem, thus triggering more careful, detailed, and bottom-up processing and attempts to resolve the perceived problem (Clore and Huntsinger, 2007; Huntsinger et al., 2014). Although feeling better/worse than forecasted is not the same as positive/negative feelings, evidence for the proposed effect would extend research by suggesting that like positive/negative feelings, feeling better/worse than forecasted may also be attention getting, have an impact on information processing, and have greater informative value on decision-making (Pham, 2004). Moreover, the principle of ‘ecological rationality’ in decision making emphasizes the importance of individual adaptation to the environment, and sees decision making as the process of using information about the environment to achieve valuable outcomes (Gigerenzer, 1998). We expected that people with forecasting bias would lead people to propose less generous offers in the positive event condition, and more generous offers in the negative event condition.

### Materials and methods

#### Participants

The participants were 192 undergraduate student volunteers at a university in central China who received course credit for participating (92 male and 100 female), with a mean age of 19.41 years (*SD* = 1.29). None of the participants had participated in the affective forecasting experiment or the ultimatum game before.

#### Procedure

The participants were invited to the laboratory, with computers presenting the E-prime 2.0 version of the ultimatum game. After signing a consent form, all of the participants were told in detail how to play the ultimatum game. All participants were randomly assigned to be allocators; 96 participants to the positive event condition and 96 participants to the negative event condition.

#### Affective forecasting bias

##### Affective forecast

The affective forecasting bias induction procedure was the same as in study 1. Before playing the game, all participants predicted their emotional reactions to both outcomes before playing the game. In a balanced manner, half of the participants were first asked to imagine that the offer was accepted, that they got the money accordingly, and then rate their anticipated emotion on the positive emotional intensity scale. They were then asked to imagine that the offer was rejected, that they got nothing, and then rated their anticipated emotion on the negative emotional intensity scale. The other half of the participants made their predictions in the opposite order.

##### Affective experience

Participants then played the ultimatum game and proposed an offer to another participant. In the positive event condition, the participants received feedback that their offers were accepted, and they got the money accordingly. They then rated their experienced emotion on the positive emotional intensity scale. In the negative event condition, on the other hand, the participants received feedback that their offers were refused, and they got nothing. Then they rated their experienced emotion on the negative emotional intensity scale.

#### Ultimatum offers

After the affective forecasting bias induction, the participants took a short break before playing a second round of the ultimatum game. All participants received instructions as follows: “The rules for this round are the same as for the previous round, so please propose an allocation to your partner from another room for the 100 RMB. Please note that if the recipient accepts the offer, you will get the money accordingly; if the recipient rejects the offer, neither of you will get the money.” The participants proposed offers to other participants. The allocation offer was rated on a 9-point scale, with 1 representing 10 RMB, 2 representing 20 RMB, 3 representing 30 RMB, and 9 representing 90 RMB.

After finishing the ultimatum game, the participants were thanked and debriefed. They were then paid according to the results of their distribution. All materials and procedures were approved by the Institutional Review Board (IRB) of the authors’ institution.

### Results

#### Manipulation checks of affective forecasting bias

As in study 1, to make within-subject comparisons of anticipated and experienced emotion, we first determined whether each participant’s offer was accepted (positive outcome) or rejected (negative outcome). We then selected the affective forecasting that corresponded to each outcome, and calculated the affective forecasting bias of participants by subtracting their ratings of experienced emotions from those of anticipated emotions.

##### Accuracy of affective forecasting

Forecasters accurately believed that their offers being accepted or rejected would make them happy or unhappy, but they were wrong about how happy (unhappy) they would be. As shown in Table 2, participants anticipated experiencing high levels of positive affect about a positive outcome (*M* = 6.94 vs. 6.34, *t* (95) = 4.64, *p* = 0.000, mean difference = 0.60, Cohen’s *d* = 0.47), and participants anticipated experiencing high levels of negative affect about a negative outcome (*M* = 6.01 vs. 5.52, *t* (95) = 4.01, *p* = 0.000, mean difference = 0.49, Cohen’s *d* = 0.41). In short, forecasters’ estimates of their affective reactions to allocation decision showed evidence of the affective forecasting bias both for positive and negative events.

#### The effect of affective forecasting on ultimatum offers

**Figure 1:**
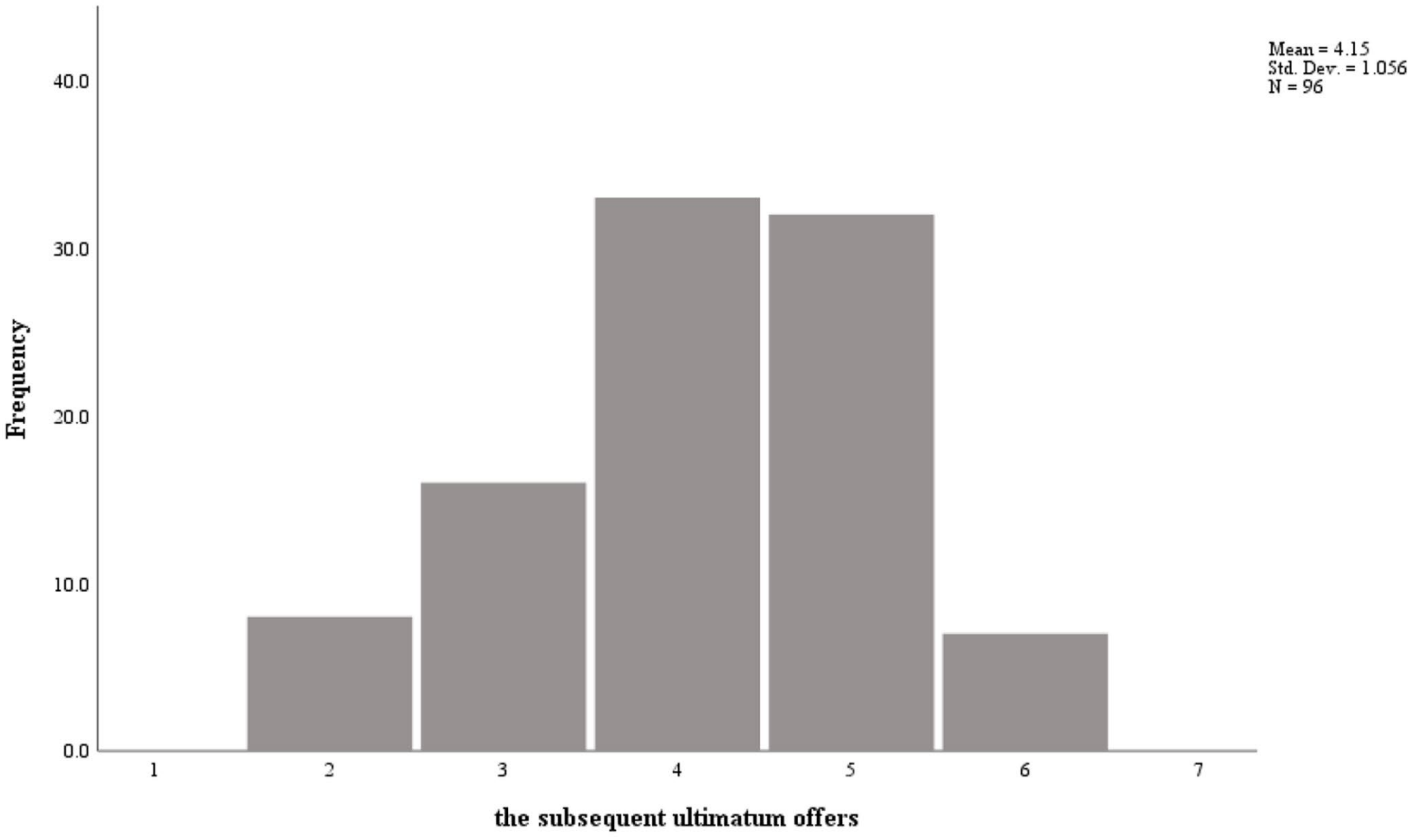
The data distribution for the subsequent ultimatum offers in positive condition.

**Figure 2:**
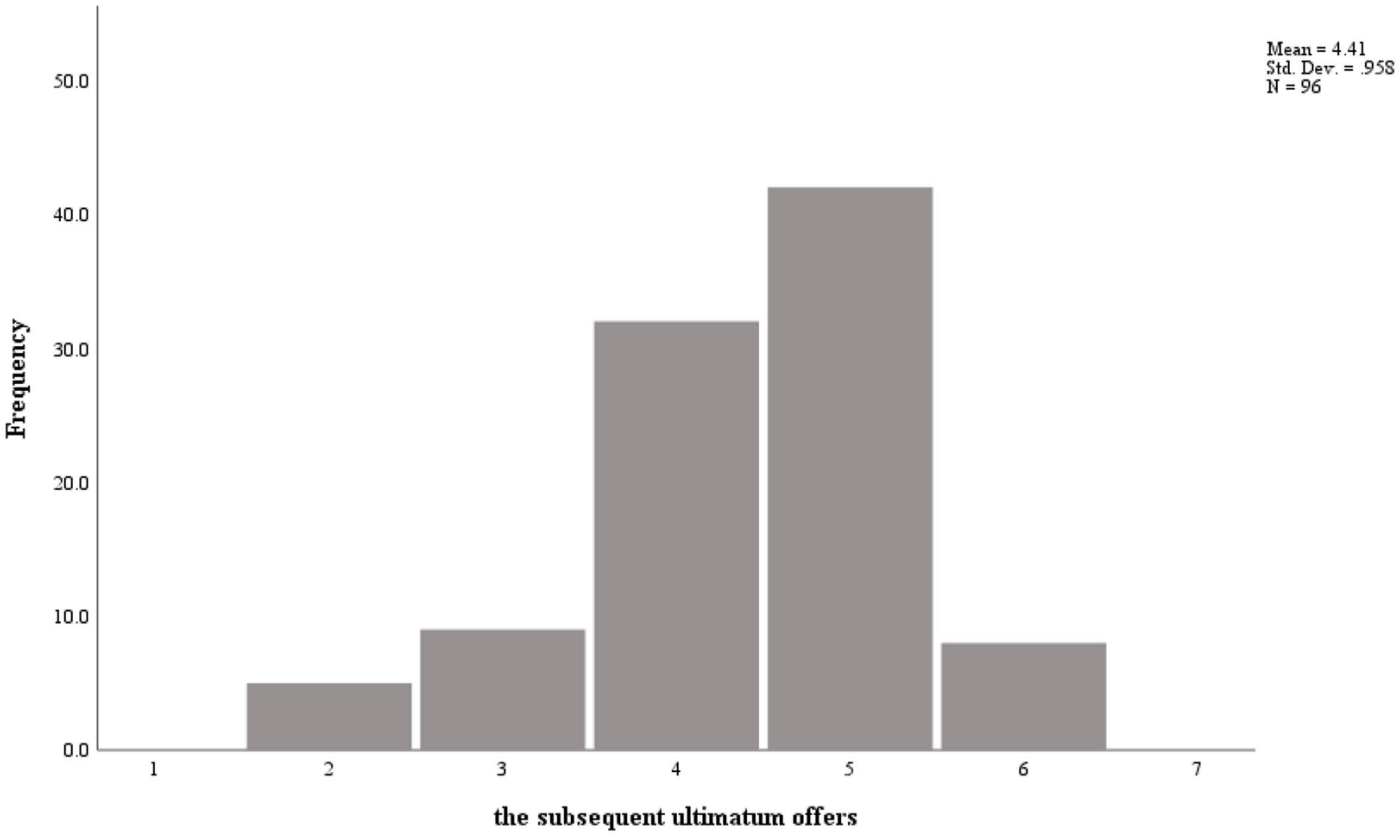
The data distribution for the subsequent ultimatum offers in negative condition.

The distribution of the subsequent ultimatum offers is shown in Figures 1, 2. In the positive event condition, the average proposed offer was 41.5 RMB (*SD* = 1.06); in the negative event condition, the average proposed offer was 44.1 RMB (*SD* = 0.96). The descriptive statistics and correlations between variables are exhibited in Tables 3, 4. As shown in Tables 3, 4, gender, age, and initial emotion were unrelated to the subsequent ultimatum offers. However, the first round offers were positively related to the subsequent ultimatum offers in the positive and negative event conditions (*r* = 0.62, *p* = 0.000 and *r* = 0.45, *p* = 0.000, respectively).

**Table 3:** Descriptive statistics and correlations between variables in the positive situation.

	M	SD	1	2	3	4	5	6	7	8
1. Gender	0.52	0.50	–							
2. Age	19.24	2.31	−0.05	–						
3. Initial emotion	6.12	1.32	0.06	−0.19	–					
4. Anticipated emotion	6.94	1.09	0.13	−0.07	0.10	–				
5. Experienced emotion	6.34	1.16	0.08	−0.02	0.09	0.38^***^	–			
6. The first round offers	4.56	0.97	0.02	−0.01	0.07	−0.03	−0.00	–		
7. Affective forecasting bias	1.17	0.75	0.00	−0.02	0.02	0.33^**^	−0.34^**^	0.29^**^	–	
8. The second round offers	4.15	1.06	0.01	−0.04	−0.07	0.07	−0.00	0.62^**^	−0.33^**^	–

*N* = 96. For Gender, 0 = male; 1 = female. * *p* < 0.05. ** *p* < 0.01. *** *p* < 0.001.

**Table 4:** Descriptive statistics and correlations between variables in the negative situation.

	*M*	*SD*	1	2	3	4	5	6	7	8
1. Gender	0.52	0.50	–							
2. Age	19.38	1.39	−0.12	–						
3. Initial emotion	3.03	1.59	0.09	0.01	–					
4. Anticipated emotion	6.01	1.80	0.14	−0.12	0.19	–				
5. Experienced emotion	5.52	1.86	0.09	−0.06	0.20*	0.79^***^	–			
6. The first round offers	4.65	0.92	0.11	0.09	−0.03	−0.10	−0.10	–		
7. Affective forecasting bias	1.08	0.70	0.08	0.07	−0.02	−0.09	−0.23^*^	0.22^*^	–	
8. The second round offers	4.41	0.96	0.10	0.03	−0.12	−0.04	−0.16	0.45^***^	0.32^**^	–

*N* = 96. For Gender, 0 = male; 1 = female. * *p* < 0.05. ** *p* < 0.01. *** *p* < 0.001.

To examine whether participants’ affective forecasting bias predicted their subsequent allocation offers in the positive event condition, we regressed participants’ allocation offers on their affective forecasting bias. Participants’ affective forecasting bias significantly predicted their subsequent allocation offers, *b* = −0.466, *SE* = 0.138, *t* (95) = −3.39, *p* = 0.001, such that the larger the affective forecasting bias, the lower the amount of money allocated to the recipient (i.e., the offers were less generous). This relationship remained strong even after controlling for the first round offer, *b* = −0.237, *SE* = 0.118, *t* (95) = −2.01, *p* = 0.047.

To examine whether participants’ affective forecasting bias predicted their subsequent allocation offers in the negative event condition, we regressed participants’ allocation offers on their affective forecasting bias. Participants’ affective forecasting bias significantly predicted their subsequent allocation offers, *b* = 0.439, *SE* = 0.133, *t* (95) = 3.29, *p* = 0.001, such that the larger the affective forecasting bias, the higher the amount of money allocated to the recipient (i.e., the offers were more generous). This relationship remained strong even after controlling for the first round offer, *b* = 0.319, *SE* = 0.125, *t* (95) = 2.55, *p* = 0.012.

### Discussion

Study 2 further replicated the affective forecasting bias in allocation decisions and provided initial evidence in support of our hypothesis that individuals’ overestimation of their future emotions affects subsequent allocation decision making. Specifically, the affective forecasting bias has a significant negative effect on allocation decisions in the positive event condition: as the affective forecasting bias increases, people are less generous and offer less money to the other person. However, the affective forecasting bias has a significant positive effect on allocation decisions in the negative event condition: as the affective forecasting bias increases, people are more generous and offer more money to the other person. These results suggest the affective forecasting bias may have implications for behavior preferences. The affective forecasting bias in the positive conditions make individuals more selfish preferences; while the bias in negative conditions make individuals more prosocial preferences.

## General discussion

Life is not always what we expect it to be, and our emotional reactions may fall short of what we expect (Buehler and Mcfarland, 2001). The results from the two studies show that there is an affective forecasting bias when forecasting the emotional outcome of resource allocation. Participants anticipated more powerful emotional reactions to both positive and negative allocation events than they end up feeling. These results provide experimental evidence for the prevalence and role of affective forecasting bias in allocation decision making. Evidence of affective forecasts bias in allocation decisions contributes to the affective forecasting literature by revealing limitations in people’s ability to assess their future feelings accurately (Feys and Anseel, 2015; Levine et al., 2018).

The present study not only shows that anticipated emotion and experienced emotion are informative in decision-making, but that the difference between anticipated and experienced emotion is also informative in allocation decisions. It demonstrates that when there is an affective forecasting bias in positive event conditions, the larger the affective forecasting bias, the less generous the decisions will be. When there is an affective forecasting bias in negative event conditions, the larger the affective forecasting bias, the more generous the decisions will be. These results extend previous findings concerning the feelings-as-information model to resource allocation and suggest that affective forecasting bias can likewise assign positive or negative values to available mental content and influence the use of different decision processing strategies in allocation decisions (Clore and Huntsinger, 2007; Huntsinger et al., 2014).

Affective forecasting bias may not necessarily lead to inappropriate decisions, but rather to different effects depending on the valence of the event. In terms of ecological rationality, affective forecasting bias in positive event conditions indicates that the experienced emotion is worse than the anticipated emotion, so individuals protect themselves by being less generous. The changed feeling indicates a decrease in emotional utility, thus individuals compensate for the outcome utility to achieve psychological balance. However, their increased uncooperative behavior will weaken their social interaction effectiveness. In negative event conditions, affective forecasting bias means the experienced emotion is better than the anticipated emotion, resulting in an increase in emotional utility that leads people to ignore monetary losses. These effects suggest that affective forecasting bias has some evolutionary adaptive implications (Gigerenzer, 1998; Dunn et al., 2007; Feys and Anseel, 2015).

The research implications above point to the fact that the way in which affective forecasts are managed in resource allocation depends on the context. In the present study, when under positive event conditions, the affective forecasting bias had a negative impact on the generosity in allocation decisions, which would undermine the effectiveness of social interaction. In this case, people’s optimistic biases may not be reasonable (Patrick et al., 2007; van Tilburg and Igou, 2019). However, when under negative event conditions, the affective forecasting bias implies overly dire negative forecasts of their future. As noted previously, people’s decisions are often guided by their anticipated affective reactions to future events. Such overly negative forecasts may undermine individuals’ motivation (Levine et al., 2018). Unrealistic positive expectations about positive events and overly dire negative forecasts about negative events may be a detrimental factor in people’s emotional well-being and life quality (Buehler and Mcfarland, 2001; Peters et al., 2014). Therefore, the results of the present study suggest that being more cautious in forecasting positive outcomes and more optimistic in forecasting negative outcomes can be beneficial to one’s well-being.

This article focuses on the relationship between affective forecasting bias and allocation decision-making, which has certain theoretical and practical implications, but there are still research limitations. The study only discusses the influence of affective prediction bias on allocation decisions, and does not address the issue of the influence mechanism. There may be some important mediating mechanisms between affective forecasting bias and allocation decisions. For example, in the positive event condition, the increase in affective forecasting bias may lead to a relative sense of loss that makes individuals to pay more attention to their personal feelings and make less generous decisions (Martinez et al., 2011; Palermo, 2017). This affective forecasting bias may also stimulate a sense of helplessness and apprehension, such that individuals may appear internally focused and egocentric, leading them to focus more on their own interests, and behave less generously (Van Kleef et al., 2006). While in the negative event condition, the increase in affective forecasting bias may lead to a relative feeling of elation that compensates for the material loss (van der Schalk et al., 2012) and makes individuals pay more attention to the feelings of others and make more altruistic decisions (Lane, 2017). Those good feelings may also cause individuals to adopt a holistic processing strategy that is more considerate of the feelings and interests of others (Huntsinger et al., 2014). Future research should empirically explore these hypothesized mechanisms.

Furthermore, the contingency factors that moderate the relationship between affective forecasting bias and allocation decisions also need to be examined in the future. Some studies have found that the effect of emotion on decision-making varies according to individual differences. For example, individual interpersonal orientation moderates the relationship between emotion and gift-giving decisions (Hooge, 2017). In addition to the individual differences’ factors, motivational factors may also play a moderating role in affective forecasting and allocation decisions (Buehler et al., 2007; Christophe and Hansenne, 2016; Pauketat et al., 2016). The moderating role of time may also need to be tested. The extant research has indicated that coping mechanisms in psychological immune system may help proposers reconcile the bias of forecasting (Gilbert et al., 1998). We might expect, for instance, that the psychological immune system would diminish the impact of forecasting bias on allocation decisions over time, as it allows proposers to reconstruct their affective forecasts and experiences. Identifying the conditions that moderate the relationship between affective forecasting and allocation decisions is also an important future research direction.

## Conclusion

Although the ability to forecast the future is one of the most prominent human abilities (Gilbert and Wilson, 2007; Miloyan and Suddendorf, 2015), not all forecasts are accurate (Wilson and Gilbert, 2003). Therefore, studying affective forecasting bias is as critical as studying affective forecasting itself. The present study demonstrated that affective forecasting bias affects allocation decisions differently in positive and negative event conditions, and helps people better understand the effect of affective forecasting on decision making. The specific findings are as follows: (1) there is an affective forecasting bias in allocation decisions, where people anticipate more powerful emotional reactions to both positive and negative allocation events than they actually experience when the events occur. (2) The increased affective forecasting bias results in less generous decisions in positive event conditions and more generous decisions in negative event conditions.

## Data availability statement

The raw data supporting the conclusions of this article will be made available by the authors, without undue reservation.

## Ethics statement

The studies involving human participants were reviewed and approved by the Institutional Review Board (IRB) of the authors’ institution. The patients/participants provided their written informed consent to participate in this study.

## Author contributions

LL contributed to experimental design, data analysis and interpretation, and writing of the manuscript. WS contributed to data interpretation and writing of the manuscript. PF contributed to experimental design and data interpretation. YJ and LT contributed to data collection and data analysis. All authors contributed to the article and approved the submitted version.

## Funding

This work was supported by the National Social Science Foundation of China [BBA160046].

## Conflict of interest

The authors declare that the research was conducted in the absence of any commercial or financial relationships that could be construed as a potential conflict of interest.

## Publisher’s note

All claims expressed in this article are solely those of the authors and do not necessarily represent those of their affiliated organizations, or those of the publisher, the editors and the reviewers. Any product that may be evaluated in this article, or claim that may be made by its manufacturer, is not guaranteed or endorsed by the publisher.
